# Same standards - different outcomes? Why clinical governance is essential to safe and consistent high-quality patient care: A call for a european governance alliance in HEMS

**DOI:** 10.1186/s13049-025-01421-3

**Published:** 2025-06-02

**Authors:** Johannes Strobel, Leif Rognås, Evi Steen, Dinis Reis Miranda, Jens Schwietring, Florian Reifferscheid, Simone Böbel, Marius Rehn

**Affiliations:** 1 Prehospital Emergency Medicine Board, German Society for Interdisciplinary Emergency and Acute Care Medicine (DGINA), Berlin, Germany; 2 Health, Ethics and Society Department, Faculty of Health, Medicine and Life Sciences, Maastricht University, Maastricht, The Netherlands; 3 Danish Air Ambulance, Aarhus, Denmark; 4 Instituut voor Medische Dringende Hulpverlening, Brugge, Belgium; 5 Traumacenter & Department of Intensive Care, Erasmus University Medical Center, Rotterdam, The Netherlands; 6 German Helicopter Emergency Medical Services (ADAC Luftrettung gGmbH), Munich, Germany; 7 German Air Rescue (DRF Stiftung Luftrettung gAG), Filderstadt, Germany; 8 Department of Anesthesiology and Intensive Care Medicine, University Hospital Schleswig-Holstein, Campus Kiel, Kiel, Germany; 9 Air Ambulance Department, Division of Prehospital Services, Oslo University Hospital, Oslo, Norway; 10 Institute of Clinical Medicine, University of Oslo, Oslo, Norway; 11 Norwegian Air Ambulance Foundation, Oslo, Norway

## Abstract

Europe is facing major challenges in security, health, and climate, which place increasing demands on emergency medical systems. While European cooperation is advancing in sectors like defence and energy, emergency medicine remains largely nationally or regionally structured. Despite significant differences in funding, staffing and mission profiles, HEMS systems across Europe share many similarities, especially regarding patient population and professional standards. The newly founded *European Governance Alliance in HEMS* aims to use these commonalities to foster cross-border learning and collaboration, based on the principles of Clinical Governance. Through regular international “Debrief & Discussion” (D&D) meetings and peer learning visits, the Alliance promotes a culture of open reflection, shared learning, and continuous improvement. The long-term goal is to define and implement common governance standards and establish sustainable structures for peer-based quality development. This initiative offers a first concrete step towards structured, international cooperation in European air rescue– with the aim of improving systems, not individuals.

Europe is currently facing profound challenges in the areas of security, society, demography, and health policy. The climate crisis brings more extreme weather, placing unprecedented strain on disaster response and emergency medical services (EMS).

While enhanced European cooperation is already being pursued in many sectors such as defence and energy, emergency medicine remains predominantly organised at national, regional, or even local levels. Yet it is precisely in this field that cross-border collaboration holds significant untapped potential to build more resilient and effective systems.

## Heterogeneous EMS landscape in Europe

The EMS landscape in Europe is organised very heterogeneously, with significant differences in funding, staff qualifications, equipment of EMS resources and even mission profile [1]. This makes comparability considerably more difficult.

Helicopter EMS (HEMS), is part of EMS in many countries and although differences exist, the HEMS systems in the various European countries have significant similarities [2]. In most countries HEMS is tasked to a similar patient population: seriously ill or injured patients. In addition, HEMS uses interprofessional teams of doctors and paramedics with internationally comparable high medical qualifications.

### The European governance alliance in Hems

The newly established *European Governance Alliance in HEMS* aims to utilise these similarities to overcome international boundaries and identify commonalities in both systems and care. In this way, we hope to be able to utilise synergies and, above all, learn from each other.

**Fig. 1:**
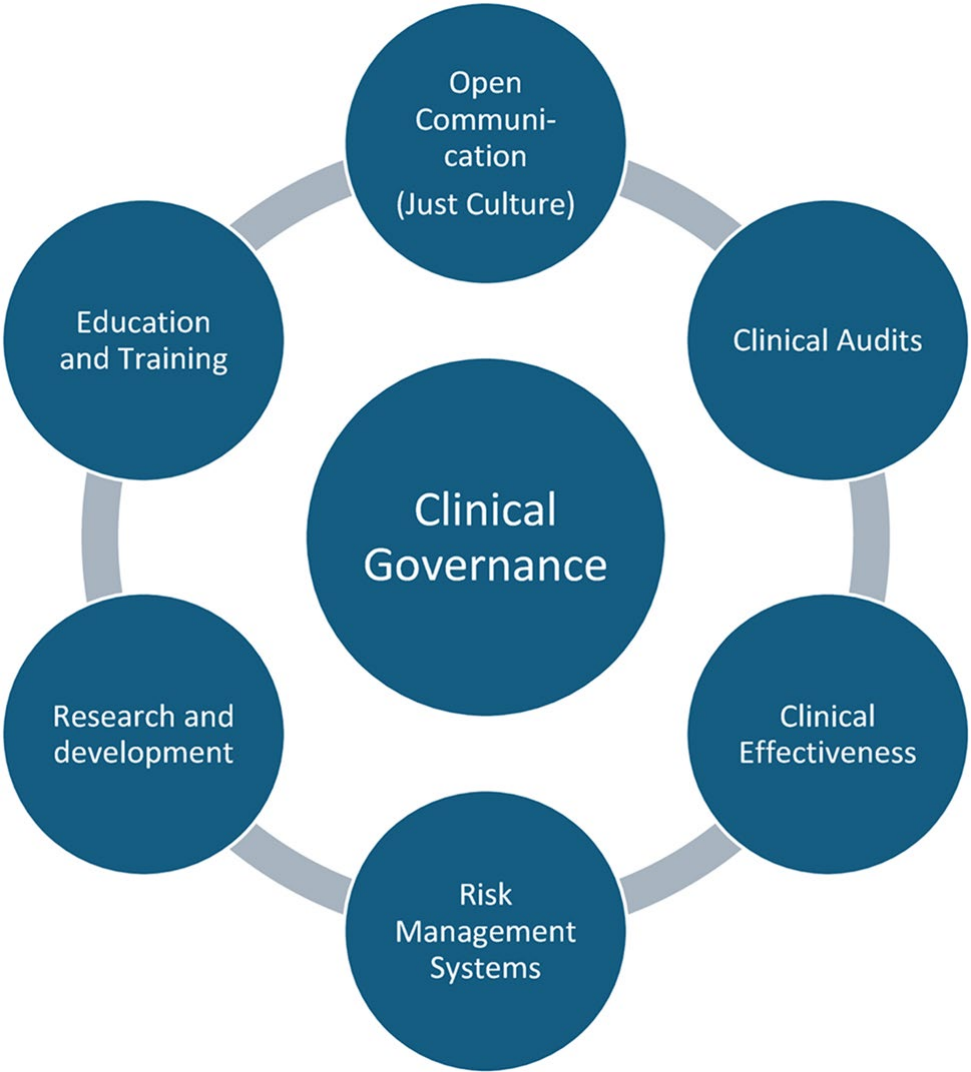
Clinical Governance in healthcare

Clinical governance (CG) in healthcare describes a framework that places patient safety at the centre to enable excellent emergency care through a continuous improvement process. CG is made up of the aspects depicted in Fig. 1 [3, 4].

This form of safety culture is particularly well established in the National Health Service (NHS) in the UK and is also embedded in UK HEMS organisations [5].

### The Idea

Clinical Audits, Clinical Effectiveness and Education & Training from this governance framework can provide a basis for establishing structured cross-border and cross-service cooperation aimed at mutual learning and the promotion of both patient safety [6] and quality of care [7].

**Fig. 2:**
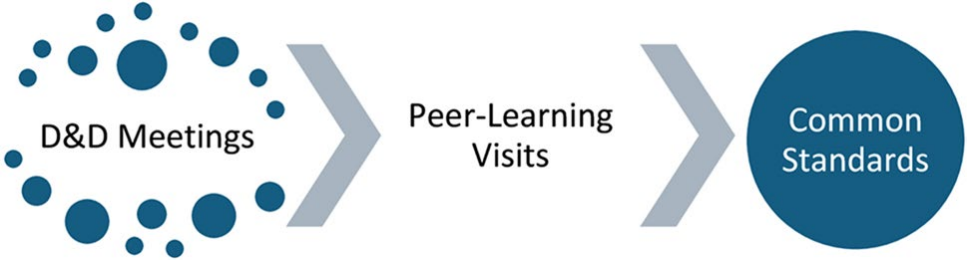
European Governance Alliance in HEMS - steps towads common Governance Standards

At the launch of the European Governance Alliance in HEMS, Senior HEMS Clinicians from Germany, Denmark, Belgium, the Netherlands and Norway met to initiate co-operation towards common Governance Standards (Fig. 2).

### International D&D meetings

As a first step, online international case conferences are to take place every two months. The aim of these ‘Debrief and Discussion (D&D) Meetings’ is firstly to establish a culture of open, curious and compassionate discussions and to explore the different approaches to real cases [8]. 

In D&D meetings, the aim is to dive deep into voluntarily selected and anonymised cases in order to explore the decision-making processes of the individual team members in the field. Goodsman and Wong describe the essence of D&D meetings as: “In medicine generally an individual’s clinical reasoning is not usually shared, let al.one as a matter of course (unless something has gone wrong). D&D serves as an opportunity for clinicians to discuss the cases and situations they encountered and hear what others might have done in the same position - potentially towards supporting (or refuting) their clinical actions and also enabling them individually and jointly to reflect on their practice.” [9].

The basic principle is not to focus on supposed mistakes, but to have a friendly discussion to identify learning points that can then be made available to all participating organisations, according to the motto “Improve the system - not the person”!

Ultimately, through this discussion platform, we can gain a deeper understanding of the differences between the European HEMS systems and EMS systems in general. Which is the basis for developing joint solutions for future challenges.

### Peer learning visits

In a second step, we suggest peer learning visits between different HEMS bases and organisations. Through peer-observation, on-site discussions and knowledge transfer, deeper insights into the organisation and the various approaches to solving common problems can hopefully be identified. As the observer is not medically active, the medical authorisation of the countries does not represent a hurdle.

In this way, representatives of the various organisations can meet independently and on an equal footing. What is important here is the willingness and motivation to learn from each other. In contrast to a certification process, the focus is not on the fulfilment of normative requirements, but on the transfer of knowledge and experience between HEMS clinicians and -systems. In this way, each organisation can hopefully become more aware of its own strengths as well as its weaknesses and vulnerabilities.

### Common governance standards

The overarching goal may be a formalised European consensus process (Delphi process). Ideally, this should define both common governance standards that not only exist in theory but have also proven themselves in practice and define the methods for an ongoing peer learning process.

Building on this, joint, cross-border work can be developed in greater depth. For example, it is conceivable to harmonise parts of the education and training offered to HEMS crews and to establish joint clinical effectiveness studies.

### The need for cross-border Cooperation

Europe is facing major challenges, and we will come up against national borders sooner than we would like. The coronavirus pandemic, for example, quickly highlighted the limits of national healthcare provision and underpinned the need for cross-border cooperation [10].

So, the question is no longer whether closer cooperation is necessary, but how quickly, how innovatively and how effectively we can implement it.

### Join Us!

The European Governance Alliance in HEMS is a first, concrete step towards structured, international cooperation in air rescue.

To make this path successful, we would like to invite other HEMS systems from Europe. Please contact us via email or LinkedIn (European Governance Alliance in HEMS) for further information. The first dates for joint D&D sessions will also be announced via LinkedIn.

**Let’s shape the future of our security architecture and, above all, air rescue together**.

## Data Availability

No datasets were generated or analysed during the current study.
